# Soybean Oil Modulates the Gut Microbiota Associated with Atherogenic Biomarkers

**DOI:** 10.3390/microorganisms8040486

**Published:** 2020-03-30

**Authors:** Hila Korach-Rechtman, Oren Rom, Lirane Mazouz, Shay Freilich, Helana Jeries, Tony Hayek, Michael Aviram, Yechezkel Kashi

**Affiliations:** 1 Laboratory of Applied Genomics, Faculty of Biotechnology and Food Engineering, Technion-Israel Institute of Technology, Haifa 3200003, Israel; krhila@campus.technion.ac.il (H.K.-R.); liranem@gmail.com (L.M.); shayfr@campus.technion.ac.il (S.F.); 2 Department of Internal Medicine, Frankel Cardiovascular Center, University of Michigan Medical Center, Ann Arbor, MI 48109, USA; roren@med.umich.edu; 3 The Lipid Research Laboratory, Rappaport Faculty of Medicine, Technion-Israel Institute of Technology, Haifa 31096, Israel; hjeries78@gmail.com (H.J.); t_hayek@rambam.health.gov.il (T.H.); aviram@technion.ac.il (M.A.); 4 Department of Internal Medicine E, Rambam Health Care Campus, Haifa 3109601, Israel

**Keywords:** microbiota, dysbiosis, soybean oil, atherosclerosis

## Abstract

During the last few decades there has been a staggering rise in human consumption of soybean-oil (SO). The microbiome and specific taxa composing it are dramatically affected by diet; specifically, by high-fat diets. Increasing evidence indicates the association between dysbiosis and health or disease state, including cardiovascular diseases (CVD) and atherosclerosis pathogenesis in human and animal models. To investigate the effects of high SO intake, C57BL/6 mice were orally supplemented with SO-based emulsion (SOE) for one month, followed by analyses of atherosclerosis-related biomarkers and microbiota profiling by 16S rRNA gene sequencing of fecal DNA. SOE-supplementation caused compositional changes to 64 taxa, including enrichment in Bacteroidetes, Mucispirillum, Prevotella and Ruminococcus, and decreased Firmicutes. These changes were previously associated with atherosclerosis in numerous studies. Among the shifted taxa, 40 significantly correlated with at least one atherosclerosis-related biomarker (FDR < 0.05), while 13 taxa positively correlated with the average of all biomarkers. These microbial alterations also caused a microbial-derived metabolic-pathways shift, including enrichment in different amino-acid metabolic-pathways known to be implicated in CVD. In conclusion, our results demonstrate dysbiosis following SOE supplementation associated with atherosclerosis-related biomarkers. These findings point to the microbiome as a possible mediator to CVD, and it may be implemented into non-invasive diagnostic tools or as potential therapeutic strategies.

## 1. Introduction

The gut microbiota, the commensal microorganisms colonizing the digestive tract, is a complex ecosystem strongly associated with host health state and well-being [1,2,3]. Host nutrition is also correlated with health state and has a predominant role in the microbiota’s composition by providing substrate availability [2,4]. High-fat diets (HFDs) have been correlated with reduced gut microbial diversity [5,6,7] as well as microbial imbalance and compositional changes, referred to as dysbiosis [7,8,9,10]. Conspicuously, fatty acid (FA) composition can also attribute to dysbiosis, while the microbiota can, in turn, modulate the host’s fatty acid composition as well as other metabolic pathways [6,11].

Atherosclerosis, an inflammatory disease of the arteries and the underlying cause of most cardiovascular diseases (CVD), was also associated with the gut microbiota [3,12,13,14]. Bacterial components (i.e., lipopolysaccharide (LPS) and peptidoglycans) as well as synthesized and released microbial-derived circulating factors, are sensed by host receptor systems and alter CVD progression [15,16,17,18,19,20,21,22,23,24,25,26,27,28,29,30,31,32,33,34].

As an outcome of the industrial processing of food, oil consumption, of soybean oil (SO) specifically, was increased dramatically during the last decades [35]. In the United States, approximately 24% of the consumed SO is used for baking and frying applications [36]. Consumers’ awareness of health issues regarding dietary fats led to soybean breeding programs designed to produce a better FA content and overall quality [36].

SO is composed of five FAs: linoleic acid (18:2) (55%), oleic acid (18:1) (18%), linolenic acid (18:3) (13%), palmitic acid (16:0) (10%) and stearic acid (18:0) (4%). This FA profile results in low oxidative stability, which limits the use of SO in food products and industrial applications [36]. Dietary FAs play a significant role in the cause and prevention of CVD; however, there are conflicting reports about the role of SO in CVD risk and atherogenesis, ranging from risk reduction, no effect or increased risk [37,38,39,40,41,42]. Herein, we aim to understand how SO modulates the gut microbiome in association with atherosclerosis-related biomarkers in mice.

## 2. Materials and Methods

### 2.1. Mouse Model and Animal Feeding

This study was carried out in accordance with the Guide for the Care and Use of Laboratory Animals of the National Institute of Health, USA. The study protocol was approved by the Committee for Supervision of Animal Experiments of the Technion—Israel Institute of Technology (Approval number: IL1411215). As previously described [42], C57BL/6 mice were chosen to study the effects of dietary SO, as this mouse strain is susceptible to developing diet-induced atherosclerosis characterized by aortic lesions and lipid-laden foam cells compared with other inbred strains (e.g., C3H and BALB/c mice) [43,44]. In addition, C57BL/6 mice do not develop atherosclerosis spontaneously as genetically altered mouse models commonly used to study atherosclerosis do (e.g., Apoe-/- or LDL receptor-/- mice) [43,44].

C57BL/6 male mice aged 6 weeks were purchased from Harlan Laboratories (Indianapolis, IN, USA). The mice were bred and housed in SPF conditions at the Animal Care Facility of the Faculty of Medicine, Technion. Mice were allowed a two weeks acclimatization period in which water and standard chow (Altromin 1324, Altromin International, Lage, Germany) containing 4.1% fat (*w*:*w*), were available ad libitum. The macronutrient and FA composition of the standard chow were previously described [42]. C57BL/6 mice readily accept SO-based emulsion (SOE) via their drinking water, and this model is commonly used in studies of fat intake by rodents [45,46]. To study the effects of SO, avoiding invasive procedures (e.g., gavage feeding or intravenous injections), the SOE was administrated to mice via their drinking water. The composition of the SOE was previously described [42].

At eight weeks of age, mice were randomly divided into the following experimental groups for a period of one month (*n* = 6–7 per group): control—no supplementation; supplementation with SOE at 80 mg/mouse/day; and supplementation at 160 mg/mouse/day. Notably, at this time point (8 weeks) the microbiome is considered to be at homeostasis, but is still susceptible to compositional changes and manipulation through diet [47]. SOE was administrated fresh daily and its complete ingestion by the mice was monitored and confirmed. Throughout the study, mice were allowed ad libitum access to chow and were weighed at baseline, after two weeks and after one month.

### 2.2. Microbiota Composition Analysis

Fecal samples were collected from each mouse preceding and following the treatment period and stored at –80 °C until processing. Total fecal DNA was extracted using QIAamp DNA stool mini kit (Qiagen, Hilden, Germany). PCR amplification of the 16S rRNA gene V3–V4 region was conducted using primers CS1-341F and CS2-806R as previously described [48]. Samples were sequenced at the DNA Services (DNAS) Facility, University of Illinois at Chicago (UIC), using a dual PCR strategy [49]. Samples were barcoded in a second PCR using the AccessArray barcoding system primer and sequenced on an Illumina MiSeq sequencer, using standard V3 chemistry with paired-end 300-base-pair reads. The resulting paired-end FASTQ files were merged using the PEAR software package. Primer sequence removal and length trimming (for sequences of <390 bp) were conducted using the software package CLC Genomics Workbench (v7; CLC Bio, Qiagen, Boston, MA, USA). Sequences were screened for chimeras using the usearch61 algorithm [50], and putative chimeras were removed from the data set. Sequence data were processed using the Quantitative Insight into Microbial Ecology (QIIME) 1.8.0 pipeline. Operational taxonomic units (OTUs) were defined based on 97% similarity clustering using the uclust algorithm [51]. Taxonomy was assigned against the Greengenes database (v13_8) as the reference [52].

### 2.3. Functional Prediction of the Microbiota

Bacterial metabolic activity abundance, as defined by the Kyoto Encyclopedia of Genes and Genomes (KEGG), were generated by Phylogenetic Investigation of Communities by Reconstruction of Unobserved States (PICRUSt version 1.1.3) [53], using closed reference OTUs picked by QIIME over the same set of sequences. Selected functions differences were compared based on the Kruskal–Wallis test using the statistical software environment R (R Foundation for Statistical Computing, Vienna, Austria).

### 2.4. Serum Analyses

As previously described [42,54,55], blood was collected from the retro-orbital plexus of each mouse under isoflurane anesthesia (via inhalation) after 16 h of overnight fasting. The serum was separated from the clotted blood by centrifugation (1000× *g*, 15 min) and kept at −80 °C. Serum cholesterol was measured using the Roche Diagnostics (Mannheim, Germany) Cholesterol Determination Kit. Serum triglycerides were measured using the Sigma-Aldrich (St. Louis, MO, USA) triglyceride determination kit, containing the T2449 triglyceride reagent, and the F6428 free glycerol reagent. As previously described [42], serum linoleic acid was measured using a Quadrupole Time-of-Flight liquid chromatography/mass spectrometry (Q-TOF LC/MS) with a 1290 infinity LC system (Agilent Technologies, Santa Clara, CA, USA) connected to C-18 reverse-phase column (XTerra C18 3.5 mm, 4.6 3 20 mm) and UHD accurate-mass Q-TOF LC/MS 6540 (Agilent Technologies, Santa Clara, CA, USA).

### 2.5. Aortic Analyses

As previously described [42,54,55], aortas were rapidly removed from the isoflurane-euthanized mice and kept at −80 °C. Subsequently, aortas were cleared of adhering fat and connective tissue and were then homogenized in 1 mL PBS using Polytron Homogenizer (Kinematica AG, Littau, Switzerland) at 60 W for 1 min. Aorta homogenates were then centrifuged (5000× *g*, 20 min) and the supernatants were analyzed for protein levels by the Lowry assay. Aortic lipids were extracted with hexane:isopropanol (3:2, *v*:*v*), and the hexane phase was evaporated under nitrogen. The amount of aortic cholesterol or triglycerides was determined spectrophotometrically using the above mentioned commercial kits. Aortic lipid peroxidation was measured by the lipid peroxide assay described by el-Saadani et al. [56]. Data were normalized to total protein levels.

### 2.6. Mouse Peritoneal Macrophages (MPM) Isolation and Analyses

As previously described [42,54,55], MPM were harvested from the peritoneal fluid of the euthanized mice, 3 days after intraperitoneal injection of 3 mL of thioglycollate (40 g/L) in saline into each mouse. The cells (2–4 × 10^7^ per mouse) were washed with PBS and centrifuged (1000× *g*, 10 min) and then resuspended in DMEM containing 1000 U/L penicillin, 100 mg/L streptomycin and 5% heat-inactivated FCS. The MPM were then plated and incubated in a humidified incubator (37 °C, 5% CO_2_) for 2 h. Then, they were washed with DMEM to remove non-adherent cells, and the monolayer was further incubated under similar conditions.

Analyses of cholesterol, triglycerides, lipid peroxides and reactive oxygen species (ROS) in MPM were performed as previously described [42,54,55]. Briefly, cellular lipids were extracted with hexane:isopropanol (3:2, *v*:*v*). The hexane phase was evaporated under nitrogen. The amounts of cellular cholesterol or triglycerides were determined spectrophotometrically using the above commercial kits. Cellular lipid peroxidation was determined by the lipid peroxide assay [56]. Data were normalized to total cellular protein measured using the Lowry assay. Intracellular ROS were measured with the 2′,7′-dichlorofluorescin-diacetate (DCFH-DA) probe using flow cytometry (BD LSRFortessa, BD Biosciences, San Jose, CA, USA).

### 2.7. Statistical Analysis

For microbiota data, diversity analyses were calculated with a rarefied OTU table containing 15,000 reads per sample. α-Diversity was calculated using Shannon’s diversity index. β-Diversity was determined by computing weighted and unweighted UniFrac distance and plotted using principal-coordinate analysis (PCoA). Differences in community composition among the different mouse groups were tested by principal coordinate (PC) score comparison based on the Kruskal–Wallis test using R. 

Linear discriminant analysis coupled with effect size measurements (LEfSe)—an algorithm for high-dimensional biomarker discovery and explanation that enables the identification of taxa characterizing the differences between two groups—was used to identify differences in taxa relative abundance within and between mouse groups and between the L3 KEGG predicted functions, using an α-value of 0.05 followed by the Kruskal–Wallis test with a threshold of 2.5 for logarithmic linear discriminant analysis (LDA) scores [57].

Bacterial taxa which showed LDA > 2.5 in the comparison between SOE treatment and control mice following the experimental period were further tested for correlation calculations with ten atherosclerosis-related markers: levels of cholesterol, triglycerides and linoleic acid in serum; MPM cholesterol, triglycerides, lipid peroxide and ROS; and aorta cholesterol, triglycerides and lipid peroxide. The Spearman correlation coefficients, *p*-values and FDR multiple corrected *p*-values were calculated using R for taxa relative abundances. The correlation with the atherogenic biomarkers markers was calculated with the null hypothesis that the mean correlation coefficient was zero, and these *p*-values were adjusted using Holm’s method.

## 3. Results

### 3.1. Microbial Composition and Diversity under SOE Supplementation

C57BL/6 mice were supplemented with 80 or 160 mg/mouse/day of SOE via their drinking water and compared with untreated control mice. Fecal samples were obtained preceding and following one month of SOE supplementation. 16S rRNA gene sequencing generated 1886,707 sequences, with an average of 30,430 reads per sample.

Overall, 586 taxa (phyla, classes, orders, families and genera) were assigned, with the majority belonging to the phyla Bacteroidetes (53–67%) and Firmicutes (26–39%).

**Figure 1:**
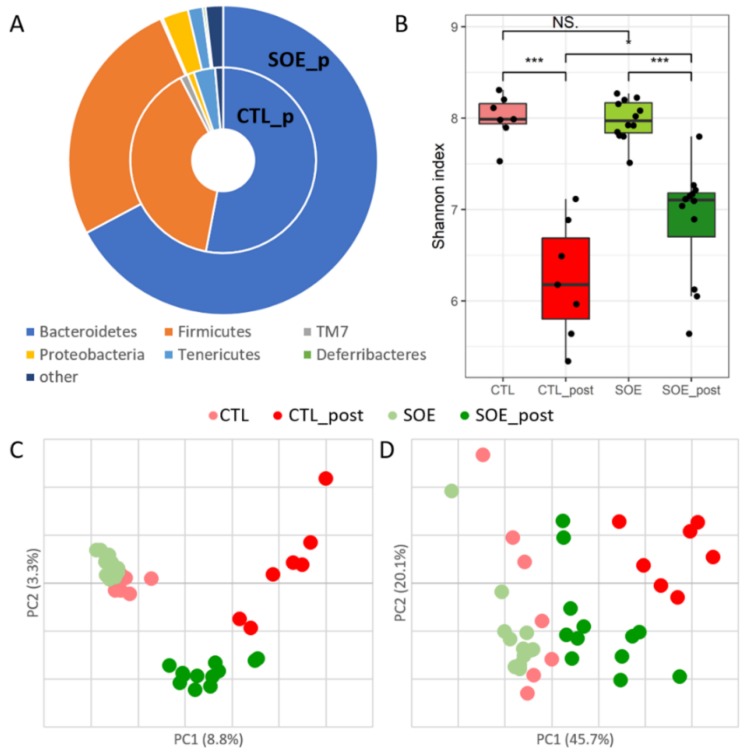
Fecal microbial composition of C57BL/6 mice is affected by soybean oil (SO)-based emulsion (SOE) supplementation. Bacterial composition was assessed by Illumina MiSeq 16S rRNA gene sequencing of fecal DNA, collected preceding (at the age of 8 weeks) and following (at the age of 12 weeks) the study period. (**A**) Phylum level relative abundance of SOE supplemented mice (inner circle) and control mice (outer circle) following the experimental period. Deferribacteres (scarcely seen in green) were found at low abundance in SOE group (0.3%). (**B**) Shannon α-diversity index of control (red) and SO (green) treated mice preceding (light colors) and following (dark colors) the experiment period. PCoA based on (**C**) unweighted and (**D**) weighted UniFrac measures. Every dot represents the entire microbiota of a single mouse. In total, 12.1% and 65.8% of total variance is explained by PCs 1 and 2 of unweighted and weighted UniFrac measures, respectively. * *p* < 0.05, *** *p* < 0.001.

Following treatment, all SOE supplemented mice showed different phylum-level abundances compared to the untreated control mice (Figure 1A). Firmicutes were less abundant following SOE supplementation, while Bacteroidetes were more abundant compared to untreated control mice (*p* < 0.002). Deferribacteres and Proteobacteria were significantly higher following SOE supplementation (*p* < 0.01), whereas Tenericutes and TM7 were significantly lower (*p* < 0.01).

The Shannon diversity index measures the within-sample diversity in each group separately [58]. As expected, we found similar richness between the groups preceding the SOE supplementation; however, decreased richness was observed following it (*p* < 0.05) (Figure 1B). The SOE supplemented mice showed higher microbial diversity than control mice following the treatment period. 

β-diversity measures the between-samples diversity and therefore represents the total microbiota composition shifts [58]. In our study, it was assessed using the phylogeny-based weighted and unweighted UniFrac measure (with and without reference to taxa relative abundance, respectively) [59]. The results were plotted using PCoA. As expected, there were no significant differences between microbiota of all experimental groups prior to the experiment period (Figure 1C,D). We observed significant separation according to the experimental groups using both UniFrac measures (*p* < 0.001) (Figure 1C,D). Principle coordinate 1 (PC1) of the unweighted UniFrac (explaining 8.8% of the variance) differentiated between all mouse groups prior to the experiment, the SOE treated mice following the experiment and the untreated control mice following the experiment. PC2 (3.3%) separates the SOE treated mice from the other samples even further. Differentiation between the groups by the weighted UniFrac appeared only by PC1 (45.7%). 

### 3.2. Key Phylotypes Shifts under SOE Supplementation

LefSe analysis was used to identify specific bacterial markers, which were shifted during the experimental period. In order to understand the experimental period’s (age) effect on the mice, we initially compared the untreated control mice preceding and following the treatment (Figure S1). Microbes which were shifted were carefully analyzed on the following analyses.

**Figure 2:**
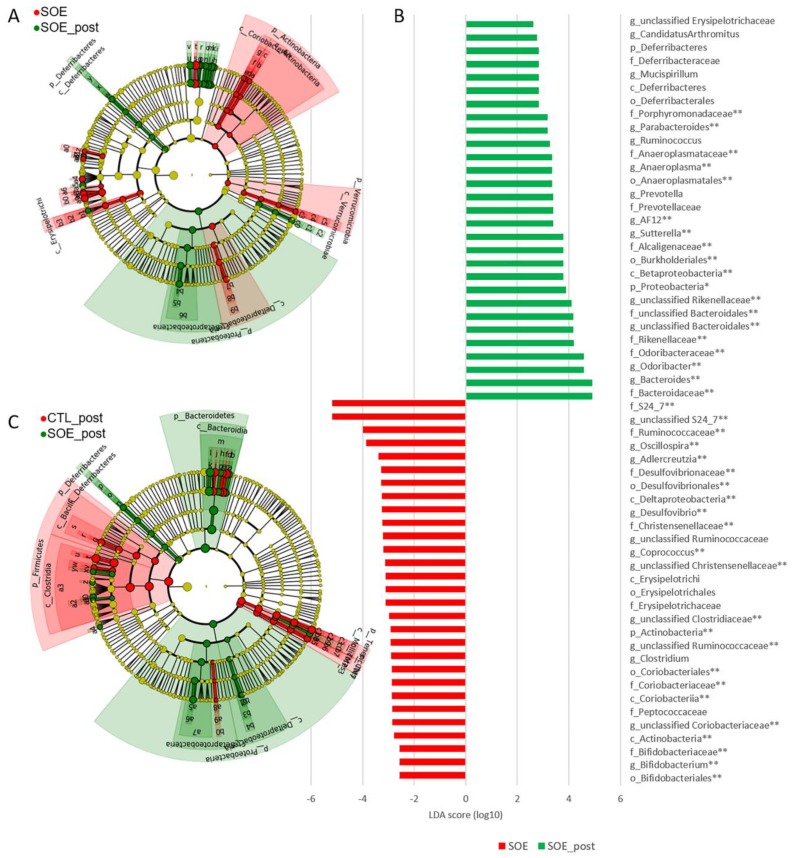
Differently abundant taxa of control and SOE treated mice. Bacterial composition was assessed by Illumina MiSeq 16S rRNA gene sequencing of fecal DNA. (**A**) Cladogram representation and (**B**) linear discriminant analysis (LDA) scores of differentially-abundant taxa between SOE supplemented mice preceding (red; 8 weeks of age) and following (green; 12 weeks of age) the study period. (**C**) Cladogram representation of differentially-abundant taxa between control (red) and SOE (green) supplemented mice following the study period (12 weeks of age). Differences were identified using LefSe and displayed in color. Each circle′s diameter is proportional to the taxa relative abundance. Only taxa meeting LDA ≥ 2.5 and *p* < 0.05 are shown. * *p* < 0.01, ** *p* < 0.005.

Comparison of SOE treated mice prior and following the experimental period revealed an increase in the prevalence of Prevotella, unclassified Rikenellaceae, Mucispirillum, Ruminococcus, unclassified Erysipelotrichaceae, Anaeroplasma and Proteobacteria (Figure 2A,B). Reduction of relative abundance was observed for unclassified Christensenellaceae, unclassified Clostridiaceae, Clostridium, Coprococcus and unclassified Ruminococcaceae. Dosage dependency had a minor effect on the microbiota’s composition, with enrichment only in Desulfovibrio for the lower SOE dosage (80 mg/day; LDA = 2.6, *p* < 0.02). Since we found such a minor dosage dependency, we addressed all SOE treated mice as one group.

**Figure 3:**
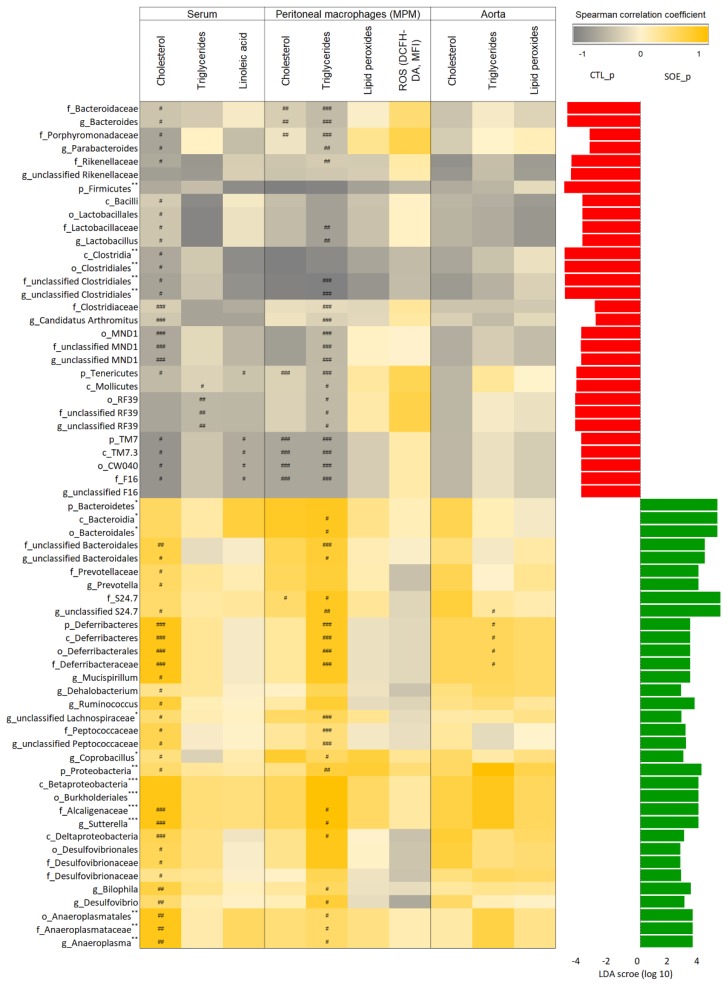
Microbial taxa altered following SOE treatment correlating with atherosclerosis-related markers (based on relative abundances). The heatmap presenting the Spearman correlation coefficient of the 64 bacterial taxa relative abundance which differed between control and SOE treated mice following the study period (sampling at the age of 12 weeks; LDA > 2.5; LDA scores appear at the right for control (red) and SOE (green)) and the atherosclerosis-related markers in serum, aorta and mouse peritoneal macrophages (MPM). Correlation coefficients are represented by colors ranging from gray (negative correlation), to yellow (positive correlation). The hashtags indicate the associations that are significant at a False Discovery Rate (FDR) of at least 20% (^#^ *p* < 0.2, ^##^ *p* < 0.1, ^###^ *p* < 0.05). The average correlation with all atherosclerosis biomarkers was tested; *p*-values were adjusted using Holm’s method and appear next to the taxa name (* *p* < 0.05, ** *p* < 0.01, *** *p* < 0.001). The biomarkers and other effects of the SOE were previously analyzed and published [42].

We also compared SOE-supplemented mice to the control mice, following the treatment period. This analysis revealed higher prevalence of Prevotella, unclassified S24-7, Mucispirillum, Dehalobacterium, Ruminococcus, unclassified Peptococcaceae, Coprobacillus, Bilophila, Desulfovibrio, Anaeroplasma and Proteobacteria in the SOE supplemented mice (Figure 2C and Figure 3). Surprisingly, few species which were age-dependent in the control group remained unchanged following SOE treatment; they included Lactobacillus (up to Bacilli class), unclassified RF39, unclassified F16 (up to TM7 phyla), Clostridiaceae family, Anaerofustis and unclassified Peptococcaceae.

### 3.3. Microbial Alteration Associated with Atherosclerosis-Related Biomarkers

Next, we evaluated the association between SOE-induced alterations in the gut microbiota’s composition and atherosclerosis-related biomarkers for each mouse. To do so, the 64 bacterial taxa which shifted following SOE treatment (LDA > 2.5) were selected and their relative abundances were correlated with the examined atherosclerosis-related biomarkers: serum cholesterol, triglycerides and linoleic acid; MPM cholesterol, triglycerides, lipid peroxides and ROS; and aorta cholesterol, triglycerides and lipid peroxides (Table S1).

After applying FDR method to account for multiple testing, we found 40 bacterial taxa abundances correlated with at least one of the biomarkers at 0.05 significance level (Figure 3); 14 of these bacteria correlated with more than one biomarker. 

Following the specific taxa correlation with a single biomarker, we aimed to reveal the average correlation between each taxa to all biomarkers. After applying Holm multiple testing correction, with 0.05 significant level, we found 18 taxa to positively or negatively correlate with all the biomarkers. The five taxa which negatively correlated with the biomarkers were more abundant in the control untreated mice following the treatment (LDA > 2.5), and included the phyla Firmicutes and unclassified Clostridiales genera up to Clostridia class. The 13 taxa which positively correlated with the biomarkers were enriched in the SOE treated mice (LDA > 2.5), and included the phyla Bacteroidetes and Proteobacteria, classes Bacteroidia and Betaproteobacteria, orders Bacteroidales and Burkholderiales, Alcaligenaceae family and genera Sutterella, Coprobacillus, an unclassified Lachnospiraceae and Anaeroplasma up to Anaeroplasmatales order.

### 3.4. Microbial Metabolic Pathways Alterations

Following the correlation with the mouse physiology, we aimed to learn the metabolic capabilities of the selected bacterial taxa. Differences in community functional attributes were evaluated using PICRUSt, which provides an avenue for functional prediction from 16S sequences. Regarding the general categories (KEGG level 1), “metabolism” was the most dominant category (~50% average) with carbohydrate metabolism (~22%) and amino acid metabolism (~21%) as the most abundant pathways (Figure S2). 

**Figure 4:**
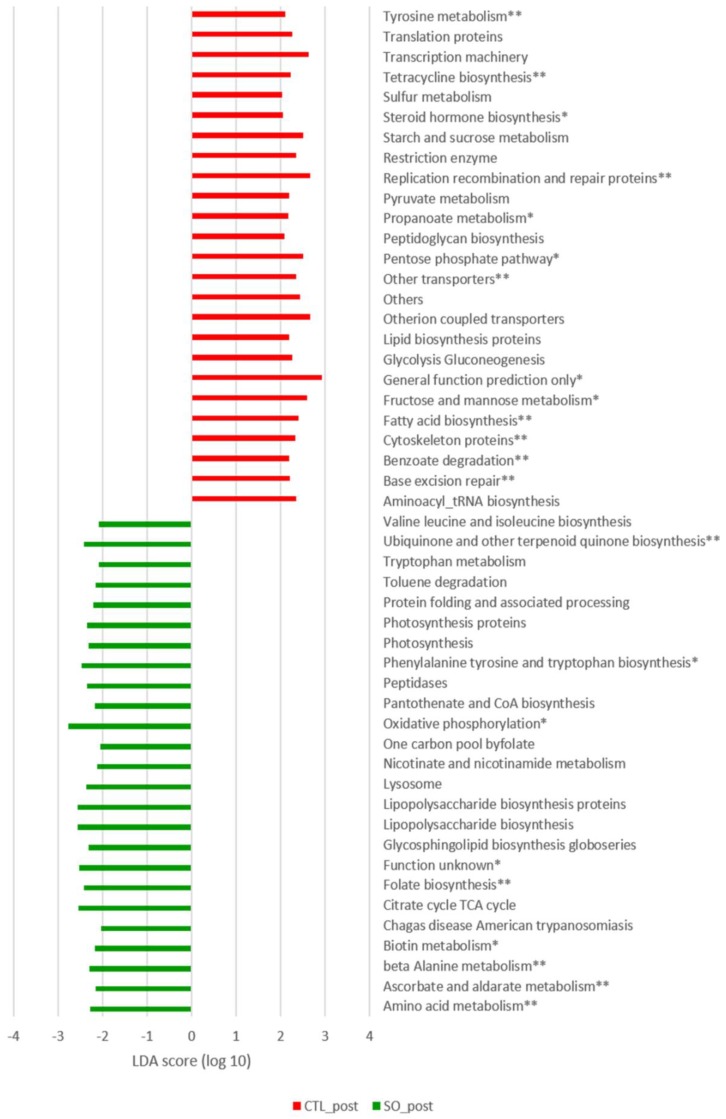
LDA scores of differently abundant inferred gut microbial metabolic pathways of control and SOE treated mice following the experimental period. Predicted microbial functions were inferred by PICRUSt from 16S rRNA gene sequences, which were assigned by closed reference Operational Taxonomic Units (OTUs) picking method. Control (red) and SOE (green) mice were compared by LEfSe analysis of KEGG based on level 3 pathways. Only pathways meeting LDA ≥ 2.5 and *p* < 0.05 are shown. * *p* < 0.01, ** *p* < 0.005.

We compared the specific functional attributes (KEGG levels 2 and 3) of control mice to the SOE treated mice following the experimental period using LefSe (Figure 4). The untreated mice showed enrichment in fatty acid biosynthesis, lipid biosynthesis proteins and carbohydrates pathways such as fructose and mannose, and starch and sucrose metabolism. The SOE treated mice showed enrichment in different amino acid metabolism, specifically valine, leucine, isoleucine, phenylalanine, tyrosine and tryptophan. Unexpectedly, lipid metabolism was not differently abundant in SOE treated mice compared to untreated control mice.

**Figure 5:**
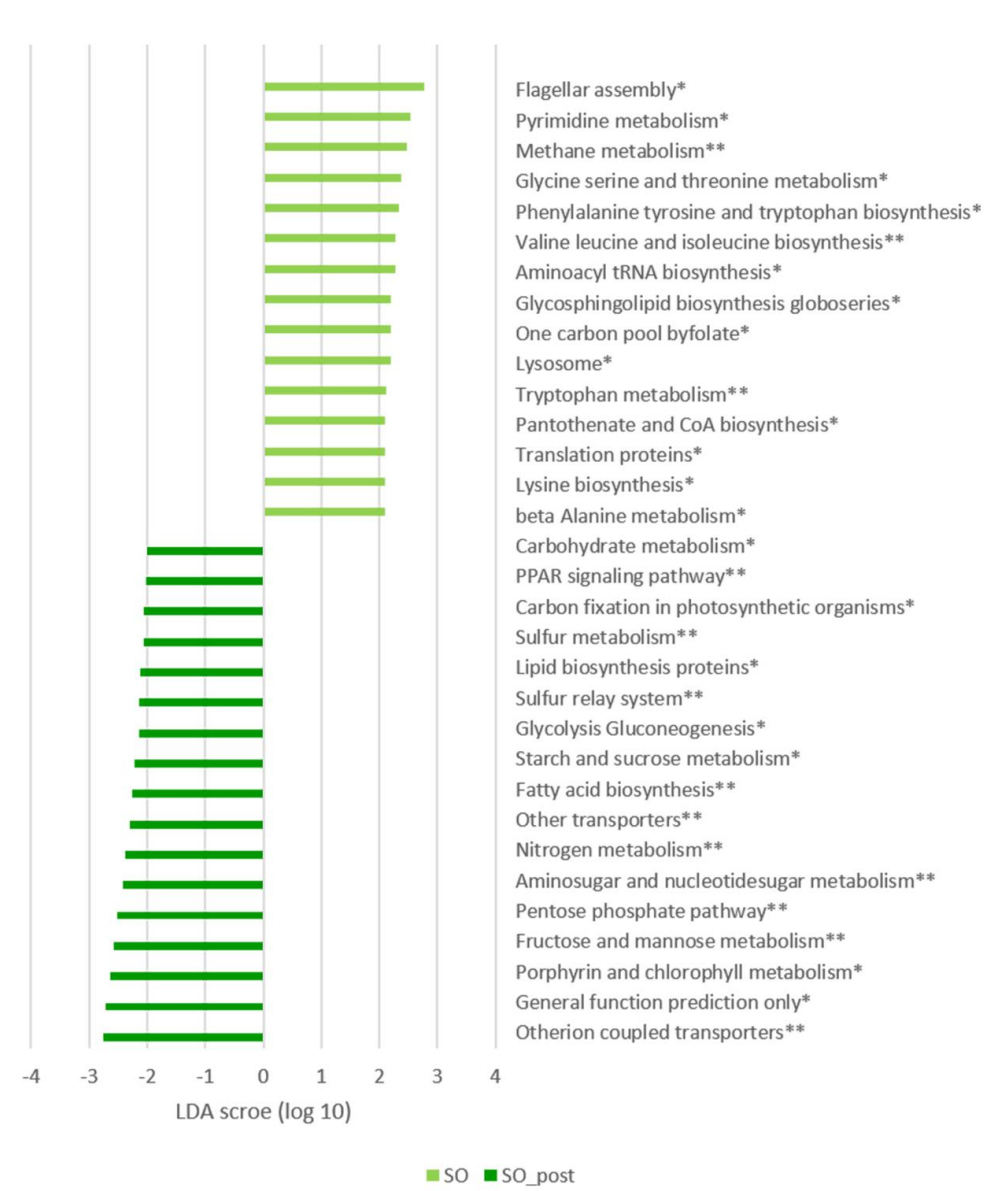
LDA scores of differently abundant inferred gut microbial metabolic pathways preceding and following SOE supplementation. Predicted microbial functions were inferred by PICRUSt from 16S rRNA gene sequences, which were assigned by closed reference OTU picking method. SOE mice preceding (light green) and following (dark green) the supplementation, were compared by LEfSe analysis of KEGG based on level 3 pathways. Only pathways meeting LDA ≥ 2.5 and *p* < 0.05 are shown. * *p* < 0.01, ** *p* < 0.005.

Comparing the SOE supplemented mice preceding and following the treatment, we observed significantly higher abundances of metabolism, cellular processes and signaling, lipid metabolism and carbohydrate predicted categories following the SOE treatment, compared to mice prior to the treatment (Figure 5).

## 4. Discussion

The association between atherosclerosis and the gut microbiota has been attracting attention, and was reported in numerus studies [3,12,13,14,20]. Herein, we aimed to characterize the microbiota and link between atherosclerosis-related biomarkers to specific microbial taxa in response to SOE consumption.

Following one month, SOE treated mice showed higher microbial richness than the control untreated mice (Figure 1B). We observed decreased microbiota diversity as age increased in all experimental groups, as previously described (Figure 1B) [5,6,60,61].

Looking at the entire microbiota, all mice at the beginning of the experiment clustered together, as expected (Figure 1C,D, Figure S1). Comparing the untreated mouse groups, we observed an age-related microbial shift (Figure 1C,D, Figure S1). These results support previous, studies indicating that the mouse age can affect the microbial population [6,47,62], albeit in a relatively short period (4 weeks). Interestingly, some of the age-related bacteria shifted in the control group, were not detected in the SOE treated mice (Figure 2). This observation is in line with a previous study reporting that HFD diminished age-related phylotypes shift [5]. Notably, the SOE effect was not caused by the total amount of fat, since we did not observe significant effect in the tested dosage.

Comparing untreated mice and SOE treated mice following the experimental period, we observed significant microbial alternations (Figure 2 and Figure 3, Figure S1), suggesting that SOE affects the gut microbiota of mice. Specifically, the SOE supplemented mice showed increase in the abundance of the phyla Bacteroidetes and Proteobacteria and a decrease in Firmicutes compared to control untreated mice (Figure 1A, Figure 2C and Figure 3). Additionally, an enrichment in Mucispirillum, Prevotella and Ruminococcus compared to untreated mice following the study period, and to the SOE group preceding the supplementation (Figure 2 and Figure 3).

Correlation analysis between bacterial abundance and atherosclerosis-related biomarkers, showed significant correlation of microbial taxa mainly with serum cholesterol level and MPM triglyceride content (Figure 3). These bacterial taxa were previously correlated with atherosclerosis and atherosclerosis-related biomarkers in human and animal studies including Prevotella [18,63], Mucispirillum [18], Anaeroplasma [18], Ruminococcus [14,54,63,64,65] and Clostridium [63,66].

Analysis of collective association between the taxa which were affected by SOE to all of the ten atherosclerosis-related biomarkers revealed 13 taxa to be positively and significantly correlated (Figure 3). Out of these taxa, several were previously reported as linked with atherosclerosis, including members of the Lachnospiraceae [14,54], Anaeroplasma [18], and Proteobacteria which was previously reported as the most abundant phylum in plaques of patients with atherosclerosis [67].

In contrast to our findings, Prevotella which was over-represented following SOE supplementation and positively correlated with serum cholesterol levels, was reported to decrease in human with atherosclerotic CVD [3]. Additionally, the observed decreased ratio of Firmicutes to Bacteroidetes, is inconsistent with other reports correlating an increase of these two phyla ratio with CVD and atherosclerosis initiation and progression in humans [67,68]. Together with other taxa in the current study, our findings are in agreement with prior reports suggesting that dysbiosis of microbes identified in a murine model are not necessarily detected in humans [18].

Dietary exposure of certain nutrients may lead to synthesis and release of microbial-derived factors, which are sensed by host receptor systems and alter CVD progression [15,16,17], including short-chain fatty acids (SCFA) [20,21,22,23,24,25], secondary bile acids (BA) [20,26,27,28,29,30,31], trimethylamine-N-oxide (TMAO) [17,18,19,20], polysaccharide-A [32], 4-ethyl phenyl sulfate [33] and catecholamines [34]. Moreover, low circulating levels of bacterial components such as lipopolysaccharide (LPS) and peptidoglycans can also activate macrophages which can reduce reverse cholesterol transport and increase insulin resistance, hyperlipidemia, and vascular inflammation [20,69,70].

As an outcome of dysbiosis, predictions of metabolic functions were also shifted. Particularly, one of the most abundant pathways that shifted following SOE supplementation was amino acid metabolism, including metabolism of the branched-chain amino acids (BCAA) valine, leucine and isoleucine as well as tryptophan metabolism. Emerging evidence indicates a significant role for dysregulated amino acid metabolism in the pathogenesis of atherosclerosis and CVD, partly via their influence on glucose and lipid metabolism as well as macrophage foam-cell formation [42,71,72,73,74]. Specifically, circulating levels of BCAA significantly and independently correlate with hyperglycemia, dyslipidemia and coronary artery diseases [75,76,77]. Herein, we found that SOE supplementation caused an increase in the abundance of the genus Prevotella. Interestingly, Prevotella copri was previously identified as the main species driving the association between biosynthesis of BCAA and insulin resistance [78].

Another metabolic pathway that was enriched in mice supplemented with SOE was tryptophan metabolism (Figure 4 and Figure 5). Microbial metabolism of tryptophan induces the production of the gut-derived uremic toxin (GDUT) indoxyl sulfate. This metabolite was shown to promote the generation of ROS in endothelial cells promoting, endothelial dysfunction and associated with aortic calcification, vascular stiffness and cardio-vascular mortality in hemodialysis patients [79].

In addition, folate biosynthesis was increased in the SOE treated mice compared to control mice following the experimental period (Figure 4). Folate plays a role in CVD due to its function in homocysteine metabolism, which may exert a direct toxin on endothelial cells, promote oxidation of low-density lipoproteins, increase DNA synthesis and promote proliferation of smooth muscle cells, or impair platelet activity [80]. The enrichment in these pathways support the studies by Rom et al. that demonstrated pro-atherogenic effects of high levels of SOE [42], and indicate that it might be mediated partly through microbial dysbiosis. 

Overall, our results demonstrate that the microbiota shift following over-consumption of SOE is not dosage dependent and not attributable to the total amount of fat. Further research is needed to reveal the specific component which causes the microbial dysbiosis, including the specific FAs in SO or the emulsifiers in the SOE. Moreover, our results indicate variation of the abundance of previously reported atherosclerosis-associated taxa and microbial derived metabolic pathways. This could be related to induction of atherosclerosis via microbiome shift. These taxa shifts may be further studied and developed to non-invasive diagnostic tool or even as potential target to therapeutic strategies.

## Supplementary Materials

The following are available online at https://www.mdpi.com/2076-2607/8/4/486/s1. Figure S1: Differently abundant taxa of control mice preceding and following the study period. Figure S2: Relative abundances of inferred gut microbial metabolic pathways of control and SOE treated mice preceding and following the experimental period. Table S1: Average atherosclerosis-related biomarkers used for correlation analyses.
