# Supplementary material for: Soybean Oil Modulates the Gut Microbiota Associated with Atherogenic Biomarkers
Source: Microorganisms. 2020 Mar 30;8(4):486. doi: 10.3390/microorganisms8040486 (PMC7232217; doi:10.3390/microorganisms8040486)
Supplement: Supplementary file 1 [file microorganisms-08-00486-s001.pdf]

Table S1: Average atherosclerosis-related biomarkers used for correlation analyses

	Control mice	SOE mice	*p* value
Serum Cholesterol (mg/dL)	101±4	128±3	<0.0005
Serum Triglycerides (mg/dL)	74±4	81±3	NS
Serum Linoleic acid (µg/ml)	192±10	224±26	NS
MPM Cholesterol (µg/mg protein)	24.2±0.5	29±1	<0.01
MPM Triglycerides (µg/mg protein)	17±1	34±3	<0.0005
MPM Lipid peroxides (µmol/mg protein)	1.0±0.1	1.1±0.1	NS
MPM ROS (DCFH-DA, MFI)	35±1	38±3	NS
Aorta Cholesterol (µg/mg protein)	4.8±0.8	11±1	<0.005
Aorta Triglycerides (µg/mg protein)	8.3±0.8	13±1	<0.05
Aorta Lipid peroxides (µmol/mg protein)	0.19±0.03	0.46±0.07	<0.05

Values are expressed as mean value ± SEM. NS – not significant.

The table was reproduced with permission from Rom et al., BioFactors, 2016 (ref. 42).
© John Wiley and Sons.

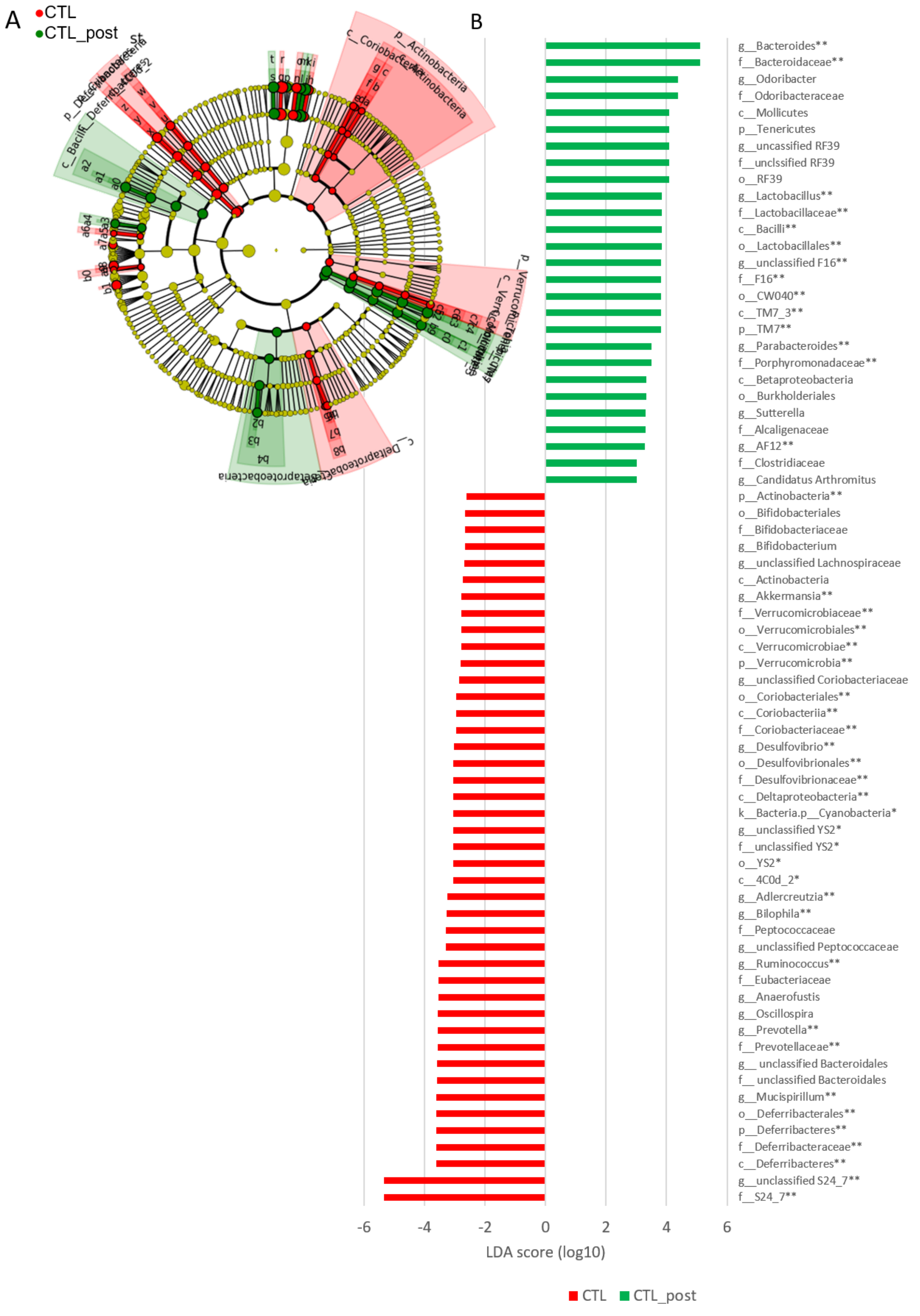

Figure S1: Differently abundant taxa of control mice preceding and following the study period

Bacterial composition was assessed by Illumina MiSeq 16S rRNA gene sequencing of fecal DNA. Differentially abundant taxa comparing control mice preceding (red) and following (green) the study period were identified using LefSe and displayed in color. Each circle's diameter is proportional to the taxa relative abundance. (A) Cladogram representation; (B) LDA scores. Only taxa meeting LDA ≥2.5 and p<0.05 are shown. *p<0.01, **p<0.005.

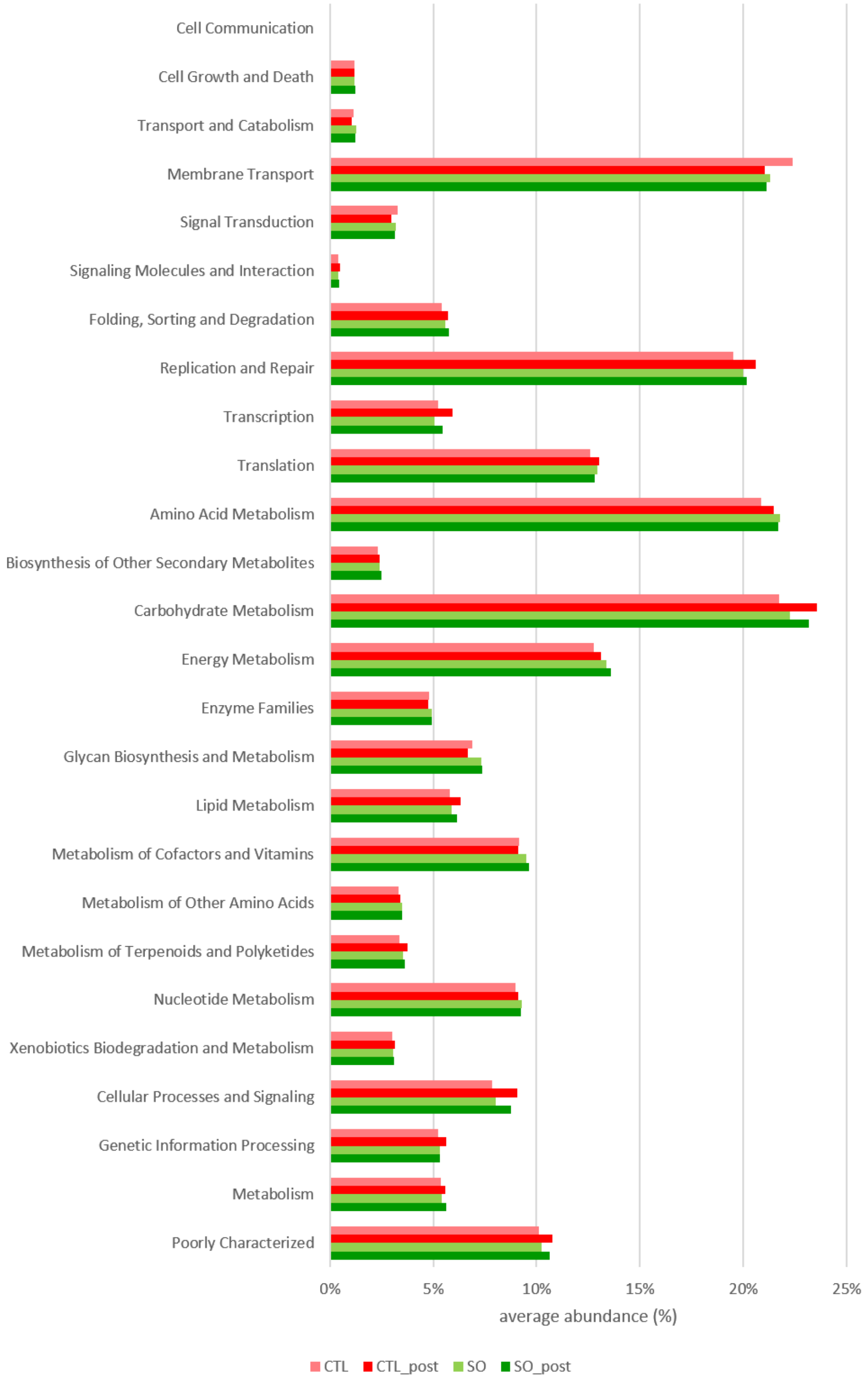

Figure S2: Relative abundance of inferred gut microbial metabolic pathways of control and SOE treated mice preceding and following the experimental period

Predicted microbial functions were inferred by PICRUSt from 16S rRNA gene sequences, which were assigned by closed reference OTU picking method. The relative abundances of level 2 KEGG functions of control (red) and SOE (green) supplemented mice preceding (light colors) and following (dark colors) the experiment period.
